# *Streptomyces* GMR22 exhibits anti-*Porphyromonas gingivalis* activity and binding affinity toward gingipain K and dipeptidyl aminopeptidase IV

**DOI:** 10.1016/j.jtumed.2026.05.010

**Published:** 2026-06-05

**Authors:** Hera Nirwati, Ema Damayanti, Metamalik Pasala, Eti N. Sholikhah, Mustofa Mustofa, Jaka Widada

**Affiliations:** a Department of Microbiology, Faculty of Medicine, Public Health and Nursing, Universitas Gadjah Mada, Yogyakarta, Indonesia; b Research Center for Food Technology and Processing, National Research and Innovation Agency, Gunungkidul, Indonesia; c Department of Pharmacology and Therapy, Faculty of Medicine, Public Health and Nursing, Universitas Gadjah Mada, Yogyakarta, Indonesia; d Department of Agricultural Microbiology, Faculty of Agriculture, Universitas Gadjah Mada, Yogyakarta, Indonesia

**Keywords:** حمض ألفا-لينولينيك, عامل مضاد للبكتيريا, البورفيروموناس اللثوية, المتسلسلة, α-linolenic acid, Antibacterial agent, *Porphyromonas gingivalis*, *Streptomyces*

## Abstract

**Objective:**

Biofilm-forming oral bacteria, particularly *Porphyromonas gingivalis*, pose serious concerns, because of their increasing antimicrobial resistance. *Streptomyces* sp. GMR22, a soil bacterium, has a large genome and is a potent antimicrobial agent. Herein, we investigated the metabolites of GMR22 responsible for antibacterial activity against *P. gingivalis*.

**Methods:**

GMR22 metabolites are produced via fermentation, extraction, and fractionation. Antibacterial assays with the microdilution method were performed on *P. gingivalis* ATCC 33277 and confirmed by scanning electron microscopy (SEM). Potentially active compounds were identified through untargeted metabolomics with ultra-high performance liquid chromatography-high-resolution mass spectrometry (UHPLC-HRMS). The binding affinities of the active compounds were evaluated through in silico molecular docking to gingipain K (KGP) and dipeptidyl aminopeptidase IV (DAP) proteins from *P. gingivalis*.

**Results:**

Two active fractions of GMR22 (F13 and F14) showed the highest antibacterial activity against *P. gingivalis*, with IC_50_ values of 20.01 and 17.44 μg/mL, respectively. SEM confirmed markedly decreased bacterial cell growth after 24 h of treatment with these fractions at 50 μg/mL under anaerobic conditions. Untargeted UHPLC-HRMS indicated that the major constituents of F13 included α-linolenic acid, β-muricholic acid, and 16-hydroxyhexadecanoic acid, whereas F14 was enriched in 2,2′-methylenebis(4-methyl-6-tert-butylphenol) and myristyl sulfate. In silico molecular docking analysis against KGP (PDB 6I9A) and DAP (PDB 2EEP) indicated that α-linolenic acid had the highest affinity (−6.92 and −7.45 kcal/mol, respectively) among all compounds (−3.38 to −5.08, respectively, and −4.08 to −6.58 kcal/mol, respectively) and native ligands (−4.75 and −4.75 kcal/mol, respectively).

**Conclusion:**

Collectively, our data identified α-linolenic acid as a major metabolite in the GMR22 fraction with in vitro anti-*P. gingivalis* activity and favorable predicted binding to biofilm- and virulence-relevant targets. *Streptomyces* sp. GMR22 metabolites therefore offer promising leads for anti-*P. gingivalis* therapeutics.

## الملخص

**أهداف البحث:**

تمثل البكتيريا الفموية المكوِّنة للأغشية الحيوية، ولا سيما البورفيروموناس اللثوية، مصدر قلق مهمًا بسبب تزايد مقاومتها لمضادات الميكروبات. وتُعد المتسلسلة GMR22، وهي بكتيريا موجودة في التربة، ذات جينوم كبير وتمتلك قدرة قوية مضادة للميكروبات. في هذه الدراسة، بحثنا في مستقلبات المتسلسلة GMR22 المسؤولة عن النشاط المضاد للبكتيريا ضد البورفيروموناس اللثوية.

**طرق البحث:**

أُنتجت مستقلبات GMR22 من خلال التخمير، والاستخلاص، والتجزئة. وأُجريت اختبارات النشاط المضاد للبكتيريا باستخدام طريقة التخفيف الدقيق على البورفيروموناس اللثوية ATCC 33277، ثم أُكّدت النتائج باستخدام المجهر الإلكتروني الماسح. وحُددت المركبات المحتمل نشاطها من خلال تحليل الأيضيات غير الموجّه باستخدام الاستشراب السائل فائق الأداء المقترن بمطيافية الكتلة عالية الدقة. كما قُيّمت ألفة ارتباط المركبات النشطة من خلال الالتحام الجزيئي المحوسب مع بروتيني جينجيباين K وثنائي الببتيديل أمينوبيبتيداز الرابع في البورفيروموناس اللثوية.

**النتائج:**

أظهر كسران نشطان من GMR22، وهما F13 وF14، أعلى نشاط مضاد للبكتيريا ضد البورفيروموناس اللثوية، بقيم تركيز مثبط نصف أعظمي بلغت 20.01 و17.44 ميكروغرام/مل، على التوالي. وأكد المجهر الإلكتروني الماسح حدوث انخفاض واضح في نمو الخلايا البكتيرية بعد 24 ساعة من المعالجة بهذين الكسرين بتركيز 50 ميكروغرام/مل في ظروف لا هوائية. وأشار تحليل الأيضيات غير الموجّه إلى أن المكونات الرئيسة في F13 شملت حمض ألفا-لينولينيك، وحمض بيتا-موريكوليك، وحمض 16-هيدروكسي هيكساديكانويك، بينما كان F14 غنيًا بمركب 2,2´-ميثيلين بيس(4-ميثيل-6-ثالثي بيوتيل فينول) وكبريتات الميريستيل. وأظهر تحليل الالتحام الجزيئي المحوسب ضد جينجيباين K وثنائي الببتيديل أمينوبيبتيداز الرابع أن حمض ألفا-لينولينيك امتلك أعلى ألفة ارتباط، إذ بلغت -6.92 و-7.45 كيلو كالوري/مول، على التوالي، مقارنةً بجميع المركبات الأخرى والربيطات الأصلية.

**الاستنتاجات:**

حددت البيانات مجتمعةً حمض ألفا-لينولينيك بوصفه مستقلبًا رئيسًا في كسر GMR22، مع نشاط مختبري مضاد للبورفيروموناس اللثوية، وارتباط متوقع ملائم بالأهداف المرتبطة بالغشاء الحيوي والضراوة. لذلك، قد تمثل مستقلبات المتسلسلة GMR22 مركبات واعدة لتطوير علاجات مضادة للبورفيروموناس اللثوية.

## Introduction

*Porphyromonas gingivalis* is an opportunistic pathogenic gram-negative bacterium associated with periodontal disease in humans.^1^^,^^2^ Antibacterial resistance to *P. gingivalis*, arising from its ability to form biofilm, remains a clinical concern. Biofilms, collections of cell masses arranged in a self-produced matrix, serve as a self-defense mechanism by increasing bacterial expression of drug resistance genes and decreasing susceptibility to antimicrobial agents.^3^ Several clinical strains of this bacterium show moderate susceptibility or resistance to amoxicillin and metronidazole.^1^ Elevated antibiotic doses are required to kill this bacterium in biofilm.^4^ Therefore, effective antimicrobial agents must be explored to treat *P. gingivalis* infections.

Gingipain K (KGP), a lysine-specific protease synthesized by *P. gingivalis*, is integral to biofilm formation, by enhancing bacterial adherence and colonization in the periodontal milieu. This enzyme's proteolytic function facilitates the breakdown of host tissues and aids in immune evasion, thereby providing an environment favorable for biofilm proliferation. Gingipains facilitate several functions that substantially contribute to bacterial survival: they initiate the kallikrein/kinin cascade; neutralize host proteinase inhibitors; promote dysregulation of coagulation and complement cascades; and degrade immunoglobulins, bactericidal proteins, and iron-transporting proteins.^5^ The enzymatic activity of dipeptidyl aminopeptidase IV (DAP) is notably high in *P. gingivalis*. Isolates with robust ability for biofilm formation have elevated DAP activity, which is greater in biofilms than in planktonic cells. Therefore, the progression of biofilm development might enhance bacterial survival and virulence via DPPIV upregulation and consequently contribute to *P. gingivalis* pathogenicity in periodontal diseases. DAP plays a critical role in the biofilm-associated virulence of this bacterium.^6^ KGP and DAP proteins, because of their critical roles in metabolism in *P. gingivalis*, might offer valuable targets for the development of anti-*P. gingivalis* therapeutic agents.

*Streptomyces* belongs to the Actinobacteria group, responsible for producing over half of the known bioactive compounds.^7^ *Streptomyces* has a large genome size of 6.2–12.7 Mb, and 5% of its bacterial genome is devoted to the synthesis of secondary metabolites.^8^^,^^9^ The exploration of new active compounds from the genus *Streptomyces* has become an important research area, because of their potential to produce novel chemical compounds that are derived from natural products and might serve as antimicrobial agents.^9^ *Streptomyces* sp. GMR22, a soil bacterium, has a genome size of 11.7 Mbp and demonstrates antifungal activity^10^, ^11^, ^12^ against species including *C. albicans*, *Fusarium oxysporum*, *Saccharomyces cerevisiae*, and *Aspergillus flavus*.^13^ Bioinformatics studies have identified that GMR22 has diverse polyketide synthase secondary metabolite-encoding genes.^12^^,^^14^

Fatty acids have been identified as active compounds in GMR22, which exhibit antifungal properties against *C. albicans*.^15^ In a preliminary study, crude extract of GMR22 has shown antibacterial activity against *P. gingivalis*.^16^ However, the GMR22 metabolites responsible for antibacterial activity against *P. gingivalis* were not determined. Several studies have reported the antibacterial properties of unsaturated fatty acids, such as α-linolenic (C_18_H_30_O_2_) acid,^17^, ^18^, ^19^, ^20^ and compounds derived from steroid acids, such as muricholic acids (C_24_H_40_O_5_).^21^ Recent research has focused on the bio-fabrication of fatty acids to enhance their potential as antibacterial, antifungal, and antibiofilm agents.^22^ However, fatty acids from Actinomycetes, particularly *Streptomyces*, have not been extensively explored, particularly as antibacterial and antibiofilm agents against *P. gingivalis*. Therefore, this study was aimed at identifying potential active compounds in *Streptomyces* sp. GMR22 that might serve as antibacterial agents against *P. gingivalis*, by using a combination of in vitro bioassays and untargeted metabolomics with liquid chromatography-high resolution mass spectrometry (LC-HRMS). In silico molecular docking studies were conducted to evaluate the binding affinity of the active compounds to KGP and DAP, which are important target proteins of *P. gingivalis*.

## Materials and methods

### Biological material

*Streptomyces* sp. GMR22 was isolated from the rhizosphere of the Cajuput plant in Wanagama Forest, Gunungkidul, Indonesia.^10^ This bacterium was deposited in the Indonesian Culture Collection under accession number InaCC A148 and in the National Biological Resource Center, Japan, under NBRC 110112. *P. gingivalis* ATCC® 33277 (Culti-loops™) was purchased from Thermo Fisher Scientific (USA) and maintained in Brain Heart Infusion (BHI) broth.

### Fermentation, extraction, and fractionation

The GMR22 isolate was maintained on ISP-2 agar medium (Difco, Sparks, USA). For fermentation, GMR22 was cultured in tryptic soy broth (Difco, Sparks, USA) as the initial medium, and 5% inoculum was added to flasks containing 500 mL starch nitrate broth at 28–29 °C with agitation at 180 rpm in a shaking incubator for 8 days.^23^ The starch nitrate broth formula and preparation procedures were as previously described.^24^ The cell-free supernatant extraction of GMR22 metabolites was performed through liquid–liquid extraction with ethyl acetate solvent, followed by evaporation with a rotary vacuum evaporator (Buchi, Switzerland).^15^ Fractionation of the solid extract was performed with flash chromatography (Reveleris™, Buchi, Switzerland), as previously described,^25^ according to the procedural manual for dry-loaded samples. The fractionation was performed with an FP SELECT C18 4 g column cartridge (Buchi); water-acetonitrile mobile phase; 15 mL/min flow rate; 3.2 min equilibration time; 25 min run length; UV1–UV4 λ = 254, 280, 366, and 600 nm, respectively; 20 mL collection peak volume; and 25 mL collection non-peak volume. All targeted fractions were weighed, and their antibacterial activity was evaluated.

### Antibacterial assays

The antibacterial activity of GMR22 metabolites against *P. gingivalis* was determined through a microdilution protocol.^26^ An initial antibacterial assay of the obtained fraction was performed at a 100 μg/mL concentration. The two identified highly active fractions were used for further antibacterial assays at serially diluted concentrations (0–125 μg/mL) in 96-well sterile microplates (Iwaki). Each well contained 100 μL BHI medium and was inoculated with 5 μL bacterial suspension (10^6^ CFU/mL). BHI medium without the active fraction served as a negative control, and BHI medium without bacteria served as a blank. The microplates were incubated at 35 °C for 24 h under anaerobic conditions (AnaeroGen, Thermo Fisher Scientific). Optical density was measured at 0 and 24 h (λ = 600 nm) with a plate reader (MultiSkan Go, Thermo Fisher Scientific). All experiments were performed in triplicate. The percentage inhibition was calculated with the following formula:

$$\%\;cell\;inhibition=(\frac{Ac-At}{Ac})\;x\;100\;$$

where *Ac* is the absorbance of the control, and *At* is the absorbance of the treatment.

Half-maximal inhibitory concentration (IC_50_) values were obtained through regression analysis of fraction concentration versus inhibition value in GraphPad PRISM 10.0.2.

### Scanning electron microscopy analysis

Scanning electron microscopy (SEM) observations were performed as previously described.^15^ *P. gingivalis* were grown at 35 °C for 24 h on sterile coverslips (22 mm diameter, SPL Life Science) in a 24-well microplate (NEST Scientific) with addition of F13 or F14 at 50 μg/mL. Cells not treated with the fractions served as a negative control. After incubation, the coverslips were washed twice with sterile phosphate buffered saline solution (Merck), dehydrated in an ethanol series (70% for 10 min, 95% for 10 min, and 100% for 20 min), and air-dried overnight. The coverslip was coated with gold through ion sputtering (Hitachi MC1000 Au ion sputter, Japan) at a setting of 10 mA for 60 s, then observed with SEM (Hitachi SU3500, Japan) at 3000 × magnification.

### Untargeted metabolomic analysis with LC-HRMS

Metabolomic analysis of the active fraction was performed with ultra-high-performance liquid chromatography (UHPLC) coupled with untargeted high-performance mass spectrometry (HRMS) (Thermo Scientific Dionex Ultimate 3000 RSLC Nano UHPLC paired with Thermo Scientific Q Exactive MS (Thermo Fisher Scientific, USA)). HRMS was performed with mobile phases A (water + 0.1% formic acid) and B (acetonitrile + 0.1% formic acid). A Hypersil Gold aQ analytical column (50 mm × 1 mm × 1.9 μm) was used with a flow rate of 40 μL/min, an injection volume of 5 μL, and an analysis time of 30 min. The gradient was programed as follows: 2 min, 5% B; 15 min, 60% B; 22 min, 95% B; 25 min, 95% B; 25.1 min, 5% B; and 30 min, 5% B. Experiments were performed in parallel reaction monitoring mode with 35,000 full width at half maximum resolution, heated electrospray ionization, positive ionization, and data processing in Thermo Scientific XCalibur.

### In silico molecular docking

The in silico testing began by determination of the optimal protein-ligand complex from the PDB. After removal of the native ligands, validation was performed by re-docking in AutoDock software. The root mean square deviation (RMSD) was calculated to verify docking accuracy. Proteins with an RMSD <2.0 Å were used for docking; otherwise, a different PDB ID was used. Grid boxes were set up in AutoDockTools to define the ligand-protein binding areas for KGP (PDB ID 6I9A) and DAP (PDB ID 2EEP) proteins. The 3D structures of the compounds were created in MolView from 2D structures, then docked to the KGP and DAP proteins. Docking was performed in AutoDockTools, with a binding energy <0 kcal/mol indicating successful ligand-protein binding. Discovery Studio was used to visualize 2D and 3D amino acid–ligand interactions. Molecular docking validation was performed by re-docking of the target protein and the respective native ligand. Validation of complexes for molecular docking was assessed with RMSD: values above the threshold of 2.0 indicated that the complex showed favorable molecular docking. However, because we were unable to identify any complex of DAP from *P. gingivalis* other than PDB ID 2EEP, we conducted molecular docking with that complex. We used H8E as the native ligand for KGP and AIO as the native ligand for DAP.

## Results

### Extraction and fractionation

**Fig. S1:**
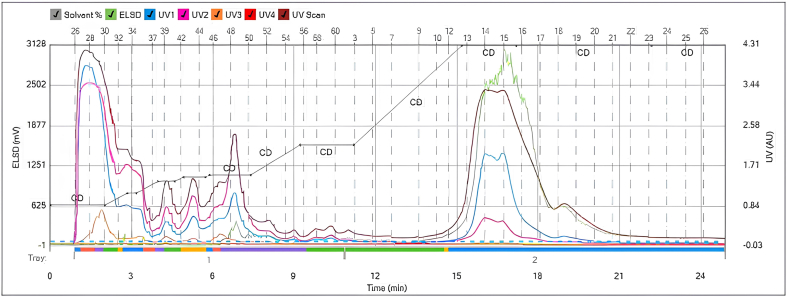
Fractionation chromatogram of *Streptomyces* sp. GMR22 extract with flash chromatography (Reveleris™), a C18 column, and water and acetonitrile as the eluent under gradient conditions.

The fractionation of the ethyl acetate extract of GMR22 with flash chromatography is shown in Supplementary Data Fig. S1. During the running time (2–6 min), high peaks were observed at UV wavelengths of 254 and 280 nm. Sample fractionation (400 mg) with flash chromatography yielded 60 tubes. Tubes with identical peaks were collected, thus resulting in 17 separate tubes. For antibacterial assays, 12 fractions with sufficient weights were selected for the initial testing.

### Antibacterial activity against *P. gingivalis*

**Figure 1:**
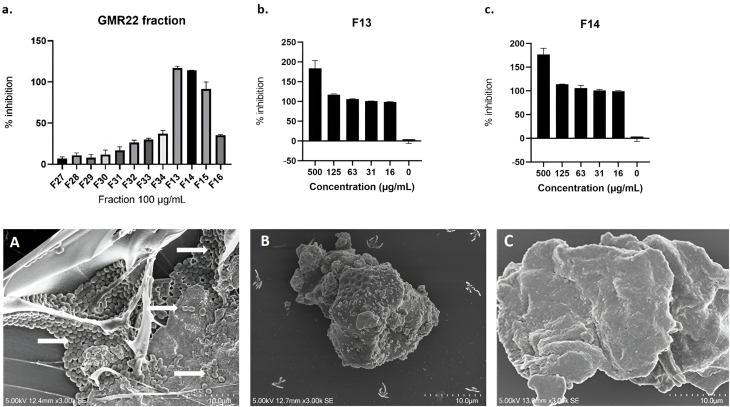
Inhibition of *Porphyromonas gingivalis* growth by the *Streptomyces* sp. GMR22 fraction (a), and fractions F13 (b) and F14 (c) containing α-linolenic acid. Scanning-electron micrograph showing decreases in *P. gingivalis* cells after treatment with the active fraction. The white arrow indicates normal cells of *P. gingivalis* in a control not treated with the active fraction (A). Decreased cell growth after treatment with the F13 (B) and F14 (C) fractions.

The antibacterial activity of GMR22 fractions was determined (Figure 1a). Two fractions (F13 and F14) showed the highest activity at 100 μg/mL (Figure 1b and 1c). The IC_50_ values of F13 and F14 against *P. gingivalis* were 20.01 and 17.44 (μg/mL), respectively. Cell growth inhibition of *P. gingivalis* by the F13 and F14 fractions of GMR22 was observed through SEM analysis (Figure 1). Administration of F13 or F14 fractions at 50 μg/mL resulted in markedly higher inhibition of *P. gingivalis* cell growth (Figure 1B and 1C) than observed in the control group without GMR22 metabolites (Figure 1A).

### Metabolite profiles

**Fig. S2:**
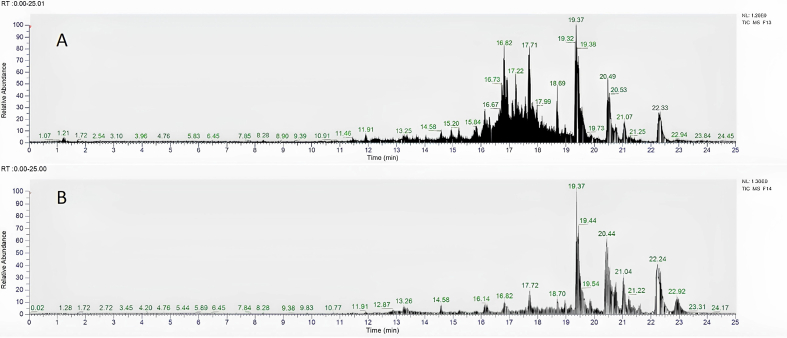
Active fraction spectra of *Streptomyces* sp. GMR22 on metabolite profiling with LC-HRMS analysis. F13 fraction (A) and F14 fraction (B).

The metabolite spectra of the active fraction obtained with LC-HRMS are shown in Supplementary Data Fig. S2, and the metabolite analyses of the F13 and F14 fractions are shown in Table 1. The detection of the major compounds of the F13 and F14 fractions at retention times of 16–23 min indicated the presence of semipolar compounds. The two fractions exhibited slightly different spectral profiles. Whereas α-linolenic acid (C_18_H_30_O_2_), β-muricholic acid (C_24_H_40_O_5_), and 16-hydroxyhexadecanoic acid (C_16_H_32_O_3_) were detected as major compounds in the F13 fraction, 2,2′-methylenebis(4-methyl-6-tert-butylphenol), myristyl sulfate, and linoleic acid were detected as major compounds in the F14 fraction.

**Table 1:** Metabolite profile of the active fraction from *Streptomyces* sp. GMR22 through untargeted LC-HRMS analysis.

No.	Name	Formula	Calculated MW	Retention Time (min)	Area (maximum) × 10^6^	mzCloud best match
	F13 fraction					
1	β-muricholic acid	C_24_H_40_O_5_	408.28731	17.477	130.19	83.9
2	16-Hydroxyhexadecanoic acid	C_16_H_32_O_3_	272.23521	19.071	109.58	88.6
3	α-Linolenic acid	C_18_H_30_O_2_	278.22453	18.702	101.25	99.9
4	Oleic acid	C_18_H_34_O_2_	282.25572	18.724	46.14	99.7
5	γ-linolenic acid	C_18_H_30_O_2_	278.22453	18.856	44.12	99.4
6	4-Heptylphenol	C_13_H_20_O	192.15053	16.076	35.68	96.2
7	2-Hydroxy myristic acid	C_14_H_28_O_3_	244.20335	17.177	30.91	98.5
8	(R)-3-hydroxy myristic acid	C_14_H_28_O_3_	244.20340	16.346	24.92	97.6
9	5-[(2Z,8Z)-2,8-Pentadecadien-1-yl]-1,3-benzenediol	C_21_H_32_O_2_	316.23997	17.951	19.01	92.3

	F14 fraction					

1	2,2′-Methylenebis(4-methyl-6-tert-butylphenol)	C_23_H_32_O_2_	340.23970	19.364	92.47	96.9
2	Myristyl sulfate	C_14_H_30_O_4_S	294.18641	16.708	81.14	94.9
3	Oleic acid alkyne	C_18_H_30_O_2_	278.22423	18.904	72.43	99.7
4	Oleic acid	C_18_H_34_O_2_	282.25584	21.815	63.68	100.0
5	α-linoleic acid	C_18_H_32_O_2_	280.23994	20.043	50.32	99.4
6	γ-linolenic acid	C_18_H_30_O_2_	278.22423	19.052	31.11	99.7
7	Labdanolic acid	C_20_H_36_O_3_	324.26619	18.832	13.43	97.5
8	Palmitic acid	C_16_H_32_O_2_	256.23987	18.234	11.45	99.4
9	16-Hydroxyhexadecanoic acid	C_16_H_32_O_3_	272.23475	16.042	9.39	98.9

### In silico molecular docking

**Figure 2:**
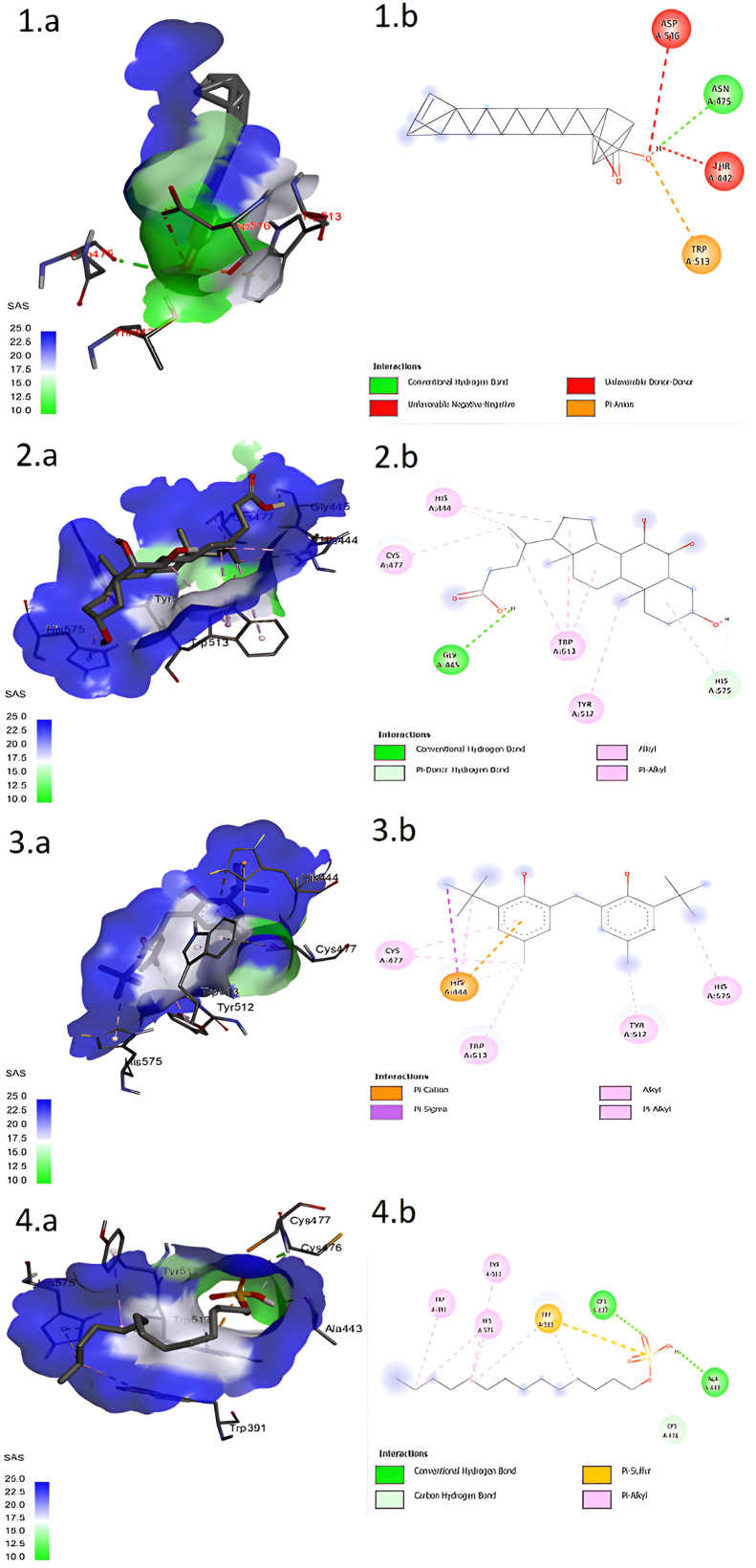
Molecular docking visualization of α-linolenic acid (1), β-muricholic acid (2), 2,2′-methylenebis(4-methyl-6-tert-butylphenol) (3), and myristyl sulfate (4) on gingipain K (KGP) protein from *P. gingivalis*. 3D (a) and 2D (b) structures.

**Figure 3:**
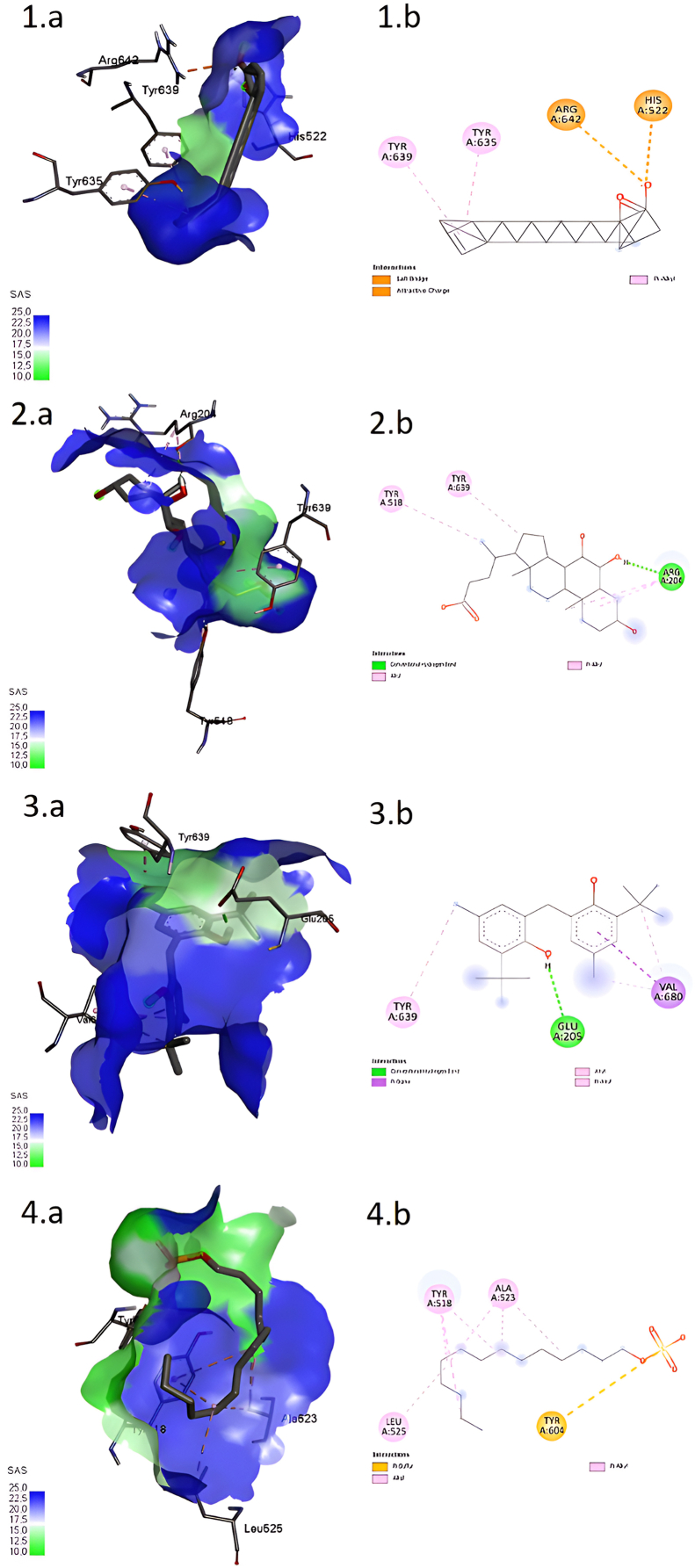
Molecular docking visualization of α-linolenic acid (1), β-muricholic acid (2), 2,2′-methylenebis(4-methyl-6-tert-butylphenol) (3), and myristyl sulfate (4) on the dipeptidyl aminopeptidase IV (DAP) protein of *P. gingivalis*. 3D (a) and 2D (b) structures.

The binding affinities of the active compounds of GMR22 toward the KGP and DAP proteins of *P. gingivalis* are shown in Table 2. On both proteins, α-linolenic acid (−6.92 and −7.46 kcal/mol, respectively), β-muricholic acid (−5.08 and −6.07 kcal/mol, respectively), and 2,2′-methylenebis(4-methyl-6-tert-butylphenol) (−5.04 and −6.58 kcal/mol, respectively) showed higher binding affinity than the other compounds and native ligands. Therefore, α-linolenic acid, the major compound in the active fraction of GMR22, exhibited the highest binding affinity toward both important *P. gingivalis* proteins. Alpha-linolenic acid showed conventional hydrogen bonding (via Asn475) and unfavorable negative–negative, unfavorable donor–donor (via Asp516 and Thr442), and π-anion (via Trp513) interactions with the KGP protein (Figure 2). Alpha-linolenic acid formed a salt bridge and π-alkyl interactions (via Tyr639 and Tyr635) with DAP, and showed charge attraction (toward Arg642 and His522) (Figure 3).

**Table 2:** Docking scores of *Streptomyces* sp. GMR22 metabolites on gingipain K (KGP) and dipeptidyl aminopeptidase IV (DAP) proteins of *P. gingivalis*, and binding amino acids.

Compounds	Affinity (kcal/mol) to KGP protein (6I9A)	Binding amino acids (KGP)	Affinity (kcal/mol) to DAP protein (2EEP)	Binding amino acids (KGP)
H8E^a^	−4,75	His444, Trp391, Trp513, Gly445, Tyr512		
α-linolenic acid	−6,92	Asp516, Asn475, Thr442, Trp513	−7,46	Tyr639, Tyr635, Arg642, His522
β-muricholic acid	−5,08	His444, Cys477, Gly445, Trp513, Tyr512, His575	−6,07	Tyr518, Tyr639, Arg204
16-Hydroxyhexadecanoic acid	−3,39	His575, Trp513, Tyr512, His444, Cys477, Ser511	−4,08	Glu205, Glu636, Tyr639, His522
2,2′-Methylenebis(4-methyl-6-tert-butylphenol)	−5,04	Cys477, His444, Trp513, Tyr512, His575	−6,58	Tyr639, Glu205, Val680
Myristyl sulfate	−4,22	Trp391, Tyr512, His575, Trp513, Cys477, Ala443, Cys476	−4,98	Tyr518, Ala523, Tyr604, Leu525
Oleic acid alkyne	−4,20	Tyr512, Trp391, Cys477, Trp513, His44, Ala443	−4,18	His522, Val680, Arg204, Tyr639, Tyr604
Oleic acid	−3,98	His444, Trp391, His575, Cys477, Trp513, Tyr512	−4,63	Tyr639, Tyr604, His710, Val680, Glu336, Gly207, Phe206
AIO^b^			−4,75	Glu205, Glu636, Tyr639, His710, Ser603

a H8E: native ligand for KGP; benzyl-N-[(2S)-1-[[(3S)-7-amino-1-(benzylamino)-1,2-dioxoheptan-3-yl]amino]-5-(2-methyl-2-phenylhydrazinyl)-1,5-dioxopentan-2-yl]carbamate (C_34_H_42_N_6_O_6_).

b AIO: native ligand for DAP; **[(**2R)-1-(L-alanyl-L-isoleucyl)pyrrolidin-2-YL]boronic acid (C_13_H_26_BN_3_O_4_).

## Discussion

Reports on the anti-*P. gingivalis* activity of *Streptomyces* are limited. One study has reported that a novel compound purified from the culture supernatant of *Streptomyces* sp. FA-70a inhibits *P. gingivalis*. Arg-gingipain, a major cysteine proteinase produced by *P. gingivalis*, exhibits virulence and survival in periodontal pockets.^27^ We observed that *P. gingivalis* biofilm formation decreased after treatment with the F13 and F14 fractions. Biofilm formation by *P. gingivalis* involves complex, dynamic, and multi-step processes. Initially, bacteria attach to abiotic or biotic surfaces (initial attachment). Subsequently, the biofilm forms a 3D structure containing microcolonies, in which the species are distinct and interact with one another (biofilm maturation). In the final phase, the cells spread out from the biofilm and form new biofilms (biofilm spreading).^3^ In this study, the GMR22 extracts were found to inhibit the initial stages (24 h) of *P. gingivalis* biofilm formation (Figure 1).

In a prior study, three muricholic acids (α-muricholic acid, β-muricholic acid, and ω-muricholic acid) have been found to inhibit germination of *Clostridium difficile* spores and to affect the growth of vegetative cells.^21^ However, 16-hydroxyhexadecanoic acid has been reported to have no antimicrobial activity,^28^ and no studies have described the antimicrobial activity of myristyl sulfate. The compound 2,2′-methylenebis(4-methyl-6-tert-butylphenol) has been reported to be an antioxidant.^29^^,^^30^ In one study, fatty acids such as α-linolenic, oleic, and linoleic acids have been found to exhibit antimicrobial activity. Linoleic acid, α-linolenic acid, and free fatty acids from the ethanol fraction of tempeh, made from fermented soybeans, have been found to exhibit antibacterial activities against *Staphylococcus aureus* and *Bacillus subtilis*.^17^ In another study, linoleic and oleic acids have shown antimicrobial and antibiofilm activity against *Pseudomonas aeruginosa*, *S. aureus*, and *Candida albicans*.^22^ A high percentage of oleic acid has been found in the nanoparticles of *Nephelium lappaceum* peel extracts, which show antibacterial activity against *Streptococcus mutans* and *S. aureus*.^31^

The mode of action of the antibacterial agent α-linolenic acid has been reported in several studies. This compound inhibits biofilm formation in *P. aeruginosa* by disrupting quorum-sensing mechanisms and decreasing virulence factor synthesis. Moreover, α-linolenic acid, administered independently or in conjunction with a complementary drug, substantially decreases biofilm mass and metabolic activity; these findings underscore its potential as a potent biofilm inhibitor and suggest that α-linolenic acid might serve as a supplementary therapeutic agent that decreases tobramycin concentrations while mitigating related adverse effects.^18^ Alpha-linolenic acid in combination with tetracycline or florfenicol has shown antibacterial effects against multidrug-resistant *Salmonella typhimurium* through several mechanisms, including inhibition of biofilm formation, disruption of the integrity of *Salmonella* cell membranes, and exocytosis pump activity.^19^ Alpha-linolenic acid effectively kills *Helicobacter pylori*, and its incorporation into liposomes increases both the attraction of liposomes to *H. pylori* and antibiotic potency.^20^ The antimicrobial activity of α-linolenic acid against *S. aureus* is associated with the fatty acid biosynthesis pathway, the main target for inhibition of bacterial metabolism.^32^

Herein, we performed in silico molecular docking to *P. gingivalis* target proteins to predict the molecular mechanisms of the active compounds detected in the GMR22 fractions. Several molecular interactions of α-linolenic acid with KGP and DAP were observed. Alpha-linolenic acid forms four ligand-receptor chemical interactions with KGP, involving Asp546, Asn475, Thr442, and Trp513, via conventional hydrogen bonding, unfavorable donor–donor interactions, unfavorable negative–negative interactions, and π-anion interactions. Unfavorable donor–donor chemical interactions occur when two donor molecules demonstrate repulsive forces attributable to their analogous electronic properties, thereby decreasing their stability and potential energy. Such interactions may obstruct the establishment of stable complexes in molecular and supramolecular chemistry, because the overlapping electron clouds of the donor molecules engage in mutual repulsion.^31^ Anion-π interactions refer to noncovalent forces between negatively charged anions and electron-deficient π-systems in aromatic compounds. These interactions originate from electrostatic attraction between the anion and the electron-rich domains of the π-cloud, which can substantially increase the binding affinity of anions to aromatic entities.^33^

Alpha-linolenic acid forms four ligand-receptor chemical interactions with DAP, including Tyr639, Tyr635, Arg642, and His522, via a salt bridge, attractive charge, and π-alkyl interactions. The π-alkyl interactions confer stability to the π-electron cloud associated with an aromatic amino acid by engaging the electron-donating characteristics of the alkyl moiety present in a separate amino acid.^34^ Salt bridges, robust noncovalent interactions between positively and negatively charged moieties, are fundamental for stabilizing protein conformation and facilitating molecular recognition. The potency of salt bridges is markedly influenced by the characteristics of the surrounding solvent medium, because solvent polarization can modify the effective interaction energy.^35^^,^^36^ The concept of attractive charges generally pertains to positive charges that interact with negative charges and consequently result in electrostatic forces. These forces are essential for shaping chemical bonds, because they stabilize the molecular architecture. This charge interaction is critical for elucidating the macroscopic properties of molecules, because it directly affects their stability and reactivity.^37^

By integrating in vitro inhibition assays, metabolomics analysis, and target-focused docking analysis, this study identified α-linolenic acid as the principal bioactive metabolite within the *Streptomyces* sp. GMR22 fraction exhibiting anti-*P. gingivalis* activity. In silico molecular docking is widely used for high-speed screening in drug discovery at the preclinical trial stage, to determine compounds’ potency and interactions with targets. Another study has used in silico molecular docking for the preclinical evaluation of spiroindimicin A-D and lynamicin A and D compounds isolated from *Streptomyces* sp. SCSIO 03032 against seven drug targets.^38^ In a recent study, in silico molecular docking has also been used to screen 15 compounds from *Streptomyces* sp. VITND1, three of which showed potential anti-bacterial, anti-oxidant, anti-inflammatory, and anti-diabetic effects.^39^

Our study has several limitations, particularly regarding the antibacterial and antibiofilm efficacy of α-linolenic acid as a single compound against *P. gingivalis*. Additionally, molecular mechanism assessment is required to validate the predictions derived from in silico molecular docking. Beyond molecular mechanisms, biofabrication of these compounds to increase activity, combination with existing therapeutic drugs, in vivo testing, and clinical trials must also be performed in future research.

## Conclusion

Our findings identified that α-linolenic acid, the main compound of *Streptomyces* sp. GMR22 showing activity against the two proteins KGP and DAP, is central to virulence against *P. gingivalis* biofilm. Furthermore, this research underscores the potential of *Streptomyces* sp. GMR22 metabolites, particularly α-linolenic acid, as promising candidates for the development of alternative anti-*P. gingivalis* strategies. We advocate for further investigation of α-linolenic acid, both as a single compound and in combination with existing therapeutic agents, as well as its molecular mechanisms as anti-*P. gingivalis* agents, to enhance potential application prospects in *P. gingivalis* therapy*.* Future research should include in vivo studies in experimental animals and clinical trials in humans to further establish the potential of α-linolenic acid as an anti-*P. gingivalis* agent.

## Ethical approval

This study was approved by the Medical and Health Research Ethics Committee of the Faculty of Medicine, Public Health, and Nursing, Universitas Gadjah Mada, Yogyakarta (ref. no. KE/FK/1576/EC/2024).

## Authors contributions

Conceptualization: HN and ED. Methodology: HN, ED, and MP. Validation: HN, MM, and JW. Formal analysis: HN, ED, MP, and ENS. Investigation: HN, ED, and MP Resources: HN and JW. Data curation: HN, ED, MM, and JW. Writing, original draft: HN and ED. Writing, review & editing: all authors. Visualization: ED and MP. Supervision: MM and JW. Project administration: HN. Funding acquisition: HN. All authors have critically reviewed and approved the final draft and are responsible for the content and similarity index of this manuscript.

## Declaration of generative AI in scientific writing

The authors state that they used a generative AI tool to correct English grammar errors in the final manuscript.

## Source of funding

This study was funded by a research assistance grant from Universitas Gadjah Mada (2024).

## Conflicts of interest

The research was conducted in the absence of any commercial or financial relationships that could be construed as potential conflicts of interest.
